# Cancer by Skin-Grafts

**Published:** 1889-02

**Authors:** 


### *CANCER BY SKIN-GRAFTS.*

In a recent number of the *Centralblatt für Chirurgie* HAHN reports a case of great pathological and therapeutical interest in which carcinomatous nodules were transplanted from one breast to the other. In this case the breast had been previously amputated for carcinoma, and was too far advanced to allow a second operation. Hahn obtained the patient's consent to ascertain if it were possible to inoculate the skin over the second breast with pieces taken from the skin of the affected mamma. Numerous small carcinomatous nodules were cut off as evenly as possible with grafting scissors, and transplanted by Reverdin's method to the sound mamma, the skin over the affected part having been removed so as to leave an open wound. The grafts took firm root by the 21st day, and the wound was completely covered by epidermis. On the 40th day some small projecting nodules, about the size of a millet seed, appeared at the edge of the pieces of skin, and gradually increased in size until by the 78th day they were as large as cherry stones. The patient died on the 82nd day. Microscopic examination of sections of the transplanted skin showed that the main mass of the tumors consisted of a well-developed connective-tissue stroma containing irregular masses of epithelial cells. These masses had insinuated themselves into the healthy tissues, which were beginning to be invaded by epithelial nests on all sides. From this case we may draw the practical deductions that carcinoma is inoculable upon healthy tissues, and that during an operation great care should be exercised to avoid taking up particles of cancerous tissue in the forceps, and leaving them adherent to the wound, where they may find a resting place and grow.

